# Comparison of the latest commercial short and long oligonucleotide microarray technologies

**DOI:** 10.1186/1471-2164-7-51

**Published:** 2006-03-15

**Authors:** Aurélien de Reyniès, Daniela Geromin, Jean-Michel Cayuela, Fabien Petel, Philippe Dessen, François Sigaux, David S Rickman

**Affiliations:** 1 Programme Cartes d'Identité des Tumeurs (CIT), Ligue Nationale Contre Le Cancer, Paris, France; 2 INSERM U462 'Lymphocyte et Cancer', Institut Universitaire d'Hematologie, Hospital Saint Louis, Paris, France; 3 Genetics and Oncology, UMR 8125 CNRS, Institute Gustave Roussy, Villejuif, France

## Abstract

**Background:**

We compared the relative precision and accuracy of expression measurements obtained from three different state-of-the-art commercial short and long-oligonucleotide microarray platforms (Affymetrix GeneChip™, GE Healthcare CodeLink™ and Agilent Technologies). The design of the comparison was chosen to judge each platform in the context of a multi-project program.

**Results:**

All wet-lab experiments and raw data acquisitions were performed independently by each commercial platform. Intra-platform reproducibility was assessed using measurements from all available targets. Inter-platform comparisons of relative signal intensities were based on a common and non-redundant set of roughly 3,400 targets chosen for their unique correspondence toward a single transcript. Despite many examples of strong similarities we found several areas of discrepancy between the different platforms.

**Conclusion:**

We found a higher level of reproducibility from one-color based microarrays (Affymetrix and CodeLink) compared to the two-color arrays from Agilent. Overall, Affymetrix data had a slightly higher level of concordance with sample-matched real-time quantitative reverse-transcriptase polymerase chain reaction (QRT-PCR) data particularly for detecting small changes in gene expression levels.

## Background

For the last decade, genome-wide expression analysis technologies have been rapidly evolving. With the growing number of large data sets derived from a variety of technological platforms (commercial and "homemade" spotted arrays) cross-laboratory studies are becoming more challenging. These kind of studies are greatly effected by differences in target type (cDNA versus short or long oligonucleotide), target production and design, the efficiencies of target labeling and sub-optimal array production/hybridization protocols [1-4]. These effects are compounded in studies involving clinically derived tissue samples such as human tumor biopsies. Efforts have been made to overcome these limitations by proposing to conform to standardize experimental conditions and procedural documentation [5]. Recently, Dobbin et al. demonstrated that by using standardized conditions and a single technological platform (Affymetrix HG-U133A), a cancer-related (RNA from tumor tissue and cell lines), multi-laboratory expression profiling study was feasible [6]. Mathematical approaches [7-10] and sequence-based mapping between platforms [11-13] have also been developed to reduce inter-technological differences in order to maximize cross-platform studies.

Nevertheless, increasing the precision and accuracy of a given microarray study will ultimately increase the yield of meaningful biological information and thus reduce the cost of genome-based biomedical discovery. Since commercial expression profiling technologies, which typically offer a higher precision compared to homemade arrays, are not only becoming more abundant but also more affordable comparative studies are useful in making a cost-efficient choice. Several of these types of studies have been published [14-22]. However, due to the rapid pace of technological development, the relevance of many of the published comparisons are quickly brought into question. Moreover, these studies have either offered conflicting conclusions or were biased by competing interests [19,22] and most have not been validated with a non-microarray-based approach, such as quantitative reverse-transcriptase PCR (QRT-PCR). Recent papers by Bammler et al. and Irizarry et al. offer a multi-laboratory comparisons of different technologies [23,24]. Bammler et al. compared 12 technological platforms and 7 different laboratories, and found that standardized protocols combined with comparisons limited to biological themes greatly increased inter-laboratory and inter-platform reproducibility. Irizarry et al. compared different dilutions of 3 cell lines analyzed at 10 different laboratories and 3 different platforms (Affymetrix oligo arrays, 2-color oligo arrays and 2-color cDNA arrays where only one platform was employed by a given lab) and found that overall level of accuracy and precision of a given technological platform are greatly affected by the differences in lab performance. Though useful, these studies do not offer a clear choice between current genome-wide, transcriptome technologies for studying human disease in the context of a centralized, multi-project (theme) program.

We present here, a comparison of the precision and accuracy of the latest and most exploited commercial high-density, pan-genomic short- and long-oligonucleotide reporter technologies. We compared data derived "in-house" from a common source of RNAs from 3 different human cell lines between Affymetrix HG-U133 plus 2.0 GeneChip™ (one-color), GE Healthcare Codelink™ (Amersham) (one-color) and Agilent Technologies 44 k whole human genome arrays (two-color). This comparison was conducted for the multi-project transcriptome program "Cartes d'Identite des Tumeurs" (CIT,[25]) launched by France's Ligue Contre le Cancer as well as for the Canceropole, Ile-de-France [26], France's cancer research network. The aim of these programs is to provide a standardized method for centralized, large-scale transcriptome analyses of a great diversity of tumors covering the maximal number of genes of interest for intra- and inter-cancer research projects. Keeping this aim in mind, we standardized the one-color with two-color data by including a common reference sample. Designs that optimize studies using two-color arrays combined with a limited number of samples (e.g. loop design, [27]) were not considered.

## Results and discussion

All raw data sets were generated by each respective commercial entity (labeling, hybridization, image analyses and pre-processing data filtering) from a common source of biologically different RNAs (MCF-7, Jurkat, and Batt P12 cell lines). Data from a pre-release version of a fourth commercial platform ("PX", one-color, 60-mer oligonucleotide-microarray) was also included in and limited to the evaluation of the level of discordance between the target measurements between the 3 platforms. The term "available targets" hereafter refers to target measurements that were not filtered out by a given platform. Different lists of targets (spotted or synthesized oligonucleotide located on the arrays) were used for the following different aspects of the analyses: all available target measurements were used for the comparisons of intra-platform reproducibility (precision); all available targets out of a common lists of 3,471 targets ("3.4 K" set) were used for all inter-platform comparisons of the accuracy of target measurement (agreement in expression levels and statistical tests results); a sub-list of 14 genes were used for the comparison of microarray data and quantitative QRT-PCR data.

### Comparison of the precision of expression measurements

**Figure 1:**
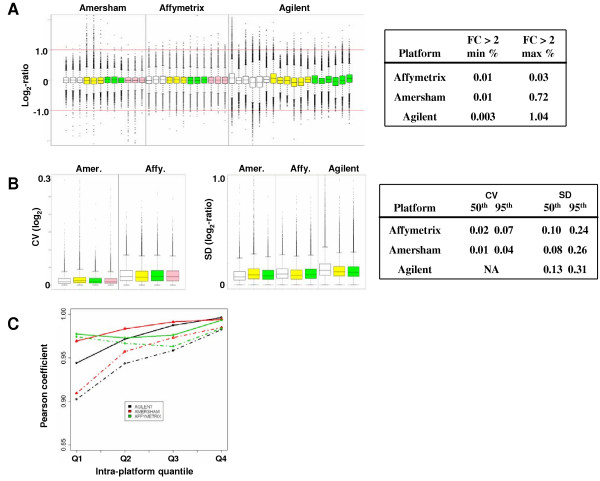
Boxplots of the distributions of the inter-slide, intra-cell line log_2_-ratios (y axis) calculated between cell line replicate pairs are shown in **A **(white: BattP12; yellow: Jurkat; green: MCF7, pink: REF). All pair-wise comparisons for each platform are shown. To the right is a summary (minimum (min %) and maximum (max %)) of the relative number of targets having a fold change superior to 2 (-1.0 > log_2_-ratio > 1.0) between replicate samples. **B **shows the box plots of the distributions of the average inter-array, intra-cell line variation measured as the coefficient of variation (CV) measured for the log_2 _intensities (Amersham and Affymetrix) or standard deviation (SD) of log_2_-ratios (Amersham (Amer.), Affymetrix (Affy.) and Agilent). To the right is a table of the 50^th ^(median) and 95^th ^percentile values obtained from different measures of variation between replicate slides (SD of log_2_-ratios and CV of log_2_-intensities). Minimum (dotted lines) and maximum (filled lines) intra-quantile correlation of expression values for each platform are shown in **C**. The values were based on log_2_-ratios. All correlation coefficients (Pearson correlation coefficient plotted on the y-axis) were calculated for all pair-wise comparisons between replicates data for the same cell line. Quantile attribution was based on the median intensities across all cell lines for a given platform where Q1 = lowest 25% mean intensities targets and Q4 = the 25% highest mean intensities targets (see Methods). Color code is as follows: green: Affymetrix; red: Amersham; black: Agilent.

In order to compare the precision of the measurements derived from each platform we used the following three criteria: 1. the percentage of targets having a 2-fold difference (or higher) of expression measurements obtained between each pair-wise combination of replicate slides; 2. the distribution of inter-slide (intra-cell line) variation measures; 3. inter-slide (intra-cell line) correlation of expression profiles analyzed for 4 intensity quantiles. One of the major observations from this study is that the data from all platforms were highly reproducible. We found that one-color platforms globally yielded lower variation measures compared to the two-color platform of Agilent (Fig. 1). We found the lowest number of 2-fold differences between replicate samples from the Affymetrix data (Fig. 1A) and the lowest CVs from Amersham. Although we found higher SDs measurements of the log_2_-ratios for one-color platforms compared to their corresponding CVs this transformation did not change the overall degree of variation relative to the two-color data obtained from Agilent. As another evaluation of intra-platform precision we calculated correlation coefficients (Pearson coefficient) stratified by intensity quantiles (Q1 to Q4, see Methods). A summary of the maximum (max) and minimum (min) correlations for a given quantile are shown in Figure 1C. We found the highest maximal level of correlation for the majority of targets from the Amersham data while Affymetrix consistently (difference between min and max) yielded a higher correlation for lower intensities (Q1 and Q2). The number of data points per quantile were not the same for each platform (roughly 12.6 K, 9.9 K and 3.9 K for Affymetrix, Agilent and Amersham, respectively), however, re-computing the correlation per quantile based on 1.6 K evenly distributed data points per quantile did not change the results (not shown). Taken altogether these results show that Affymetrix data was found to have a slightly overall higher level of precision compared to the other platforms.

### Comparison of the accuracy of expression measurements

The evaluation of platform accuracy was based on the following criteria: 1. concordance between the expression levels of commonly represented targets on each array type; 2. agreement in the identification of genes discriminating the three cell lines; 3. concordance of relative expression compared to data obtained from QRT-PCR for subset of 14 genes. The first 2 evaluations were based on the available targets from the 3.4 K set. This 3.4 K set was specifically chosen in order to reduce the confounding effects of multiplicity (N targets: transcript (RefSeq identifier) relationship, see Methods). This approach afforded the most straightforward study of the concordance across the different platforms and did not favor platforms with high feature-to-target and/or target-to-transcript ratios. Moreover, this 3.4 K set of targets is representative, at the array feature-intensity level, of overall target measurements for each array type. Roughly 25% of the available 3.4 K targets were attributed to each of the 4 intensity quantile bins for each platform (quantile bins are based on all available targets, see Methods and [Additional file 2]). Expression values above the filtering thresholds were available from all platforms for 2,218 out of the 3.4 K common targets.

#### Similarity in relative expression levels

**Figure 2:**
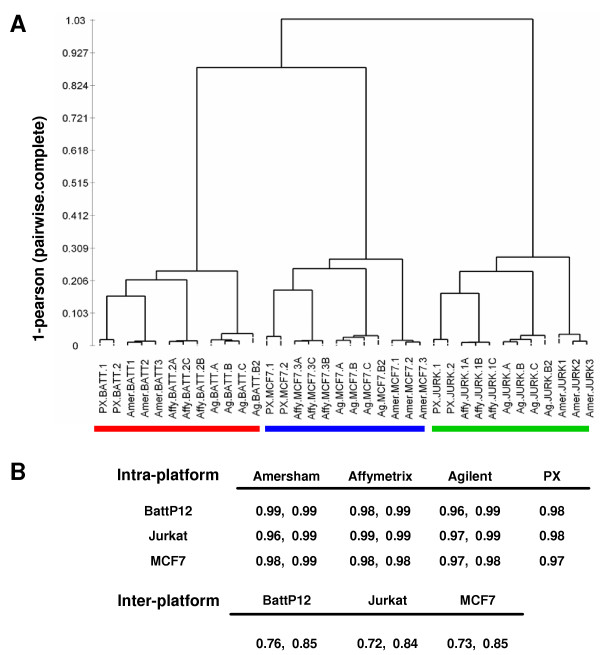
Clustering of all samples based on log_2_-ratio data of 3.4 K common transcripts is shown in **A**. The dendrogram shows three major clusters of samples indicated by red (Batt P12 samples), blue (MCF7 samples) and green (Jurkat samples). Pearson correlation coefficients corresponding to the maximum and minimum correlation nodes in the dendrogram shown in **A **(intra- and inter-platform correlations) are given in **B**. Since the platforms detected a different number of targets from slide to slide, all correlations were calculated using the maximal number of targets detected in common between each pair-wise combination (complete, pair-wise correlation).

Cluster analysis of log_2_-ratio data from all platforms yielded clusters of samples grouped according to the three cell lines showing a high overall correlation between the 4 platforms (high minimal intra-platform (0.96) and inter-platform (0.72) correlation, see Fig. 2). We observed that one of the Amersham Jurkat samples clustered slightly apart from the other two Jurkat samples, consistent with the heterogeneity in the reproducibility of the Amersham observed in Figure 1. However, visual inspection of the array images, overall distributions of the data (boxplots and MA plots, not shown) and the equal percentage of available data points (56% – 61%) for Jurkat samples relative to the other cell line samples (53% – 62%) did not provide an explanation for this observation.

**Figure 3:**
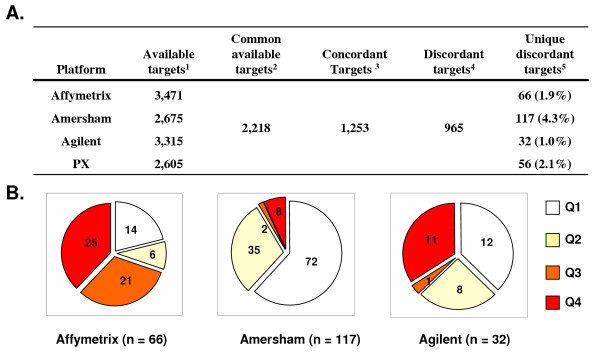
**(A) **Summary table of a comparison of the available targets out of the 3.4 K for each platform. ^1 ^The number of targets out the 3.4 K common set that were available for at least one sample for a given platform. ^2 ^The number of targets out the 3.4 K common set that were commonly available for at least one sample for all platforms. ^3^The number of targets that were attributed with a similar intensity quantiles nomenclature (identical or off by 1 quantile, see Methods) for Affymetrix, Amersham, Agilent and PX. ^4 ^The number of targets that were attributed with a different intensity quantile (off by 2 quantiles) for at least 2 platforms. ^5 ^The number of targets attributed with a different intensity quantile (off by 2 quantiles) for a given platform compared to the other 3 platforms. **(B) **The fraction of uniquely discordant targets by quantile for each platform with the following color code: white: Q1; yellow: Q2; orange: Q3; red: Q4. The numbers indicate the number of targets that fell into that particular quantile specifically for that particular platform and that should have fallen into another quantile based on the agreement of quantile attribution for those targets between the other platforms.

Nevertheless, we found a significant number of aberrant target measurements unique to particular platform. Here the data from PX was included in order to confirm the discordance in target measurement observed for a particular platform. We found that while most of the 2,218 targets had a similar overall expression values between the 4 platforms 965 targets were found to be discordant across the different platforms (Fig. 3A). Of these, we found 271 targets that were discordant uniquely for either Affymetrix, Agilent or Amersham (difference of at least 2 intensity quantile attributions between 1 platform and the other 3). Of the three platforms, Agilent contributed the lowest number of discordant expression values (32) compared to Affymetrix and Amersham (66 and 117, respectively). The discordant targets found for Agilent were dispersed evenly into three Agilent quantile bins (Q1, Q2 and Q3–4) whereas 91% of the discordant targets unique to Amersham in its lowest 2 intensity quantiles (Q1 and Q2) suggesting a problem of sensitivity (Fig. 3B). We found that 60% of the discordant targets found for Affymetrix were in its Q3 and Q4 quantiles suggesting a more fundamental problem in target identification.

Differences in target availability and discordant expression values between the platforms may be related to the differences in feature design, sequence and their placement along the target transcript. Recent sequence-based identification studies have suggested that annotations attributed to target sequence is a non-negligible source of discrepancy between technologies [13,28]. Using a sequence-based identification for Affymetrix probe sets on the HG-U133 plus 2.0 arrays Harbig et al. not only re-named over 20,000 probe sets but flagged over 5,000 probe sets that specifically hybridize to either more than one splice variants (multiplicity problem) or transcripts from different loci (specificity problem). We should note that none of the Affymetrix probe sets flagged by Harbig et al. were among the 3.4 K set. Unfortunately, comparisons between the different platforms, at this level, were not feasible as feature location and sequence information are publicly available for only Affymetrix and Agilent.

#### Agreement in the identification of differentially expressed genes

Starting from all available targets out of the 3.4 K set for each platform we performed independent ANOVA and t test analyses. From the ANOVA analyses, we obtained 1,696, 1,727 and 1,781 significant targets (p < 0.001) for Affymetrix, Amersham and Agilent, respectively (Table 1). Based on all 280 possible multivariate permutations for a given platform we found that the probability of obtaining these results by chance if there are no real differences between the classes was 0.00357. We found a lower percentage of the total amount of "ANOVA targets" (roughly 4 – 7.5%) attributed with intra-platform quantile Q1 for all platforms. Affymetrix data yielded fewer ANOVA targets (12%) in its Q1 and Q2 bins compared to the 22% and 32% found for Agilent 44 K and Amersham, respectively. We found roughly the same percentage of ANOVA targets (59–60%) that had similar levels of expression for each respective platform (consensus quantiles cQ1 to cQ4, see Methods). Of these, approximately the same low proportion (roughly 2.0%) corresponded to low-expressing transcripts (a cQ inferior to cQ2.0). Overall, these results suggest that there is a common limitation of the 3 technologies to detect differences in expression of the lowest expressing genes.

**Figure 4:**
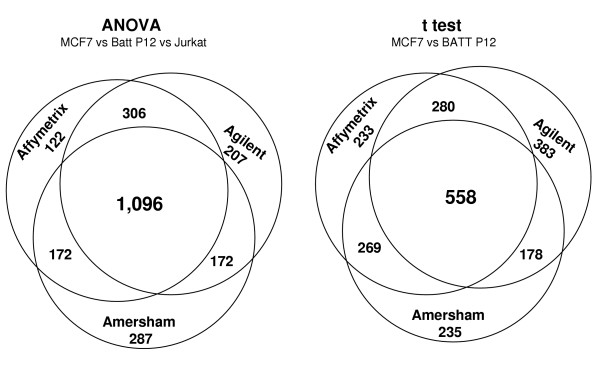
Venn diagram showing the overlap of 2,362 targets (the union of all ANOVA targets) that were considered significantly different in a F test (p < 0.001) between the cell lines calculated for at least one platform (left). It must be noted that the numbers indicating the targets unique to a platform or common to 2 platforms include targets that may have been filtered out by the other platform(s) during the preprocessing. See Table 2 for a summary of platform-unique targets that were available (not filtered) for all platforms. The Venn diagram to the right shows the overlap of the 2,135 targets discriminating MCF7 and Batt P12 samples (p < 0.001, union of all 3 platform results) for at least one platform.

Figure 4 summarizes the agreement or overlap of the ANOVA targets as well as for the targets distinguishing MCF7 and Batt P12 samples identified for the 3 platforms. A total of 2,362 and 2,135 discriminating targets were found for at least 1 out of the 3 platforms based on ANOVA and t tests, respectively. While a considerable number of targets were unique to one or two platforms we observed that 1,096 ANOVA targets were in common between all 3 platforms. Many of the targets correspond to known cancer genes such as NFkB1, BUB1B, CDK7, CCNB2, CCND3 and CXCR4. The expression profiles of the 1,096 common ANOVA targets were highly correlative between the platforms. Similar to what we observed in Figure 2, a pan-platform cluster analysis based on these targets yielded 3 major sample clusters corresponding to the 3 cell lines [see Additional file 5]. Within each of the 3 major cell line clusters, samples were grouped according to platform. Cluster analysis based on the expression profiles of the 1,096 common ANOVA targets available in a larger variety of tissue samples yielded biologically relevant sample groups, consistent with the 3 major clusters observed in Figure 4A.

Based on the ANOVA results we found the least amount of commonality from the Amersham data (Table 2). Of the 287 targets unique to Amersham, 171 were available (not filtered) for all platforms, 28 of which were expressed at high levels (not shown) and 47 yielded p values > 0.05 for both Affymetrix and Agilent. Moreover, 69% (157 out of 228 targets) of the ANOVA targets found by Agilent and Affymetrix yielded p values superior to 0.05 for Amersham. Limiting the tests to the identification of genes that discriminate MCF7 and Batt P12 samples we observed a much lower number of genes (558) common to all 3 different platforms. We also observed a higher level of discordance with the Agilent data. Taking all tests into consideration, we found a slightly higher level of agreement (least amount of discordance) in the identification of discriminating genes from the Affymetrix data.

#### Evaluation of relative expression measures compared to QRT-PCR data

In order to address the discrepancies in accuracy mentioned above we analyzed 14 genes (plus one control gene) using real-time QRT-PCR. These genes passed the pre-processing filters in at least one out all of the samples studied. In addition, these genes were selected based on either 1. complete concordance (CCNB1) or discordance between either their measured expression levels (CASP5, CXCL10, MAPK4, RAB17, RME8 and TMPRSS5, and WNT10B) and/or being identified as a discriminating gene between the different platforms (ALG8, CASP5, CXCL10, MAPK4, RAG2, THEM2, TMPRSS5); 2. three genes (ERBB2, RPS6KB1, BCAS2) known to be highly expressed in MCF7 cells [29-32]. ERBB2 and RPS6KB1 corresponded to multiple targets to a different extent for the 3 platforms and were therefore excluded from the overall correlation calculation between RT-PCR and array data as well as from the all gene-wise comparison (see below). The 14 genes covered a large dynamic range of expression levels (maximal Ct = 37.85 (low relative level of expression); minimal Ct = 20.60 (high relative level of expression), see [Additional file 4] for all array data and sample-matched QRT-PCR Ct values for the corresponding genes).

Despite a high overall correlation between array and RT-PCR data (Table 3) we found several discrepancies between the different platforms. Based on the RT-PCR data we detected 12 out of the 14 genes in all 4 samples (3 cell lines and the REF sample). RAG2 was out of the limit of detection in BattP12 and MCF7 samples while detected in Jurkat samples. RAG2 was equally filtered out for BattP12 and MCF7 in 3 out of the 4 replicate Agilent arrays and all of the replicate Amersham arrays, whereas this gene was found to be a "unique ANOVA" target for Affymetrix. CASP5 was below the level of detection in Jurkat but yielded high Ct values in BattP12 and MCF7 samples. However, for the Agilent data, CASP5 was detected by all arrays barring one BattP12 replicate array. CASP5 was considered a low expressing gene for in all Affymetrix arrays (Q2) and Amersham arrays (Q1). It should be noted that the data provided by Agilent was derived from experiments in which about 1/10 of the amount of material was used for a hybridization (0.75 μg of cRNA compared 10 μg of cRNA used by the other platforms) in accordance with their recommended protocol. As expected, RT-PCR values for ERBB2, RPS6KB1, BCAS2 were relatively higher in MCF7 compared to the other samples. Similarly, all 3 platforms yielded consistently high relative expression values for RPS6KB1 and BCAS2. Array data for ERBB2, however, were found to be equally inconsistent from all 3 platforms.

A gene by gene analysis of the expression relative to the reference sample revealed a high agreement between array data and QRT-PCR data (Table 4). This observation was independent of the overall expression level as assessed by the average Ct values for each gene. Here, 'agreement' refers to the same direction of relative expression (over or under-expressed) for a gene in a given cell line relative to the reference sample. We found that Affymetrix yielded a slightly higher overall score compared with the other 2 platforms. A recent paper studying the correlation between Affymetrix HG-U133A data (normalized using either RMA or MAS5.0) from 48 genes and QRT-PCR data found an equally high correlation (r = 0.89) with both methods for roughly 85% of the genes [33]. They found poor correlations with genes that were either expressed at low levels or those for whose probe sets were not adequately designed. Based on our analysis we did not observe an expression-level dependence on the correlation between Affymetrix, Agilent and Amersham array data and the QRT-PCR data.

**Figure 5:**
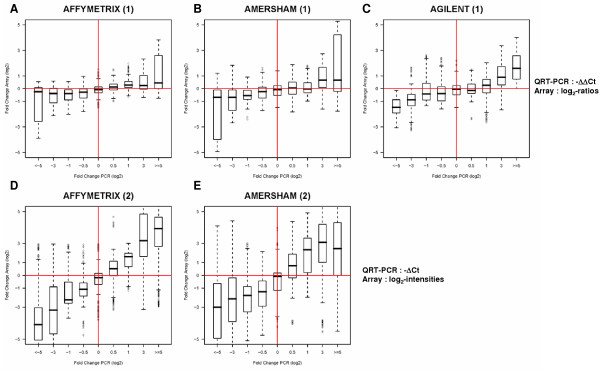
Boxplots of the distributions of all the gene pair relative expression levels calculated across all cell lines for the different platforms. From a total number of 4,851 possible gene pair combinations based on the array measurements of 12 out of the 14 genes (excluding ERBB2 and RPS6KB1 and data points (RAG2 and CASP5) not available from QRT-PCR) in the 3 cell lines (3 replicate arrays each) a total number of 3,321 and 3,828 combinations were considered for ratio and single intensity array data, respectively. See [Additional file 4] for the number of possible versus available gene pair combinations for the QRT-PCR and array data, respectively. Combinations were then attributed to one of 9 different fold-change bins based on the -ΔΔCt (for A – C) or -ΔCt (D and E) values calculated from the QRT-PCR data with the following code (in bold) corresponding the following geneA/geneB log_2_-ratios (x) intervals: **< -5**: x < -5; **-3**: -5 = x < -1.25; **-1**: -1.25 = x < -0.75; **-0.5**: -0.75 = x < -0.25; **0**: -0.25 = x < 0.25; **0.5**: 0.25 = x > 1.25; **1**: 0.25 = x > 1.25; **3**: 1.25 = x > 5.0; **> = 5**: x = 5.0. For each gene pair combination we assessed the corresponding FCs either from the (1) array log_2_-ratio (cell line/REF) data (Affymetrix, Amersham, and Agilent, shown in A – C, respectively) or from (2) log_2_-intensities (Affymetrix and Amersham data only, shown in D and E).

We extended this analysis to an all gene pair-wise comparison of relative expression levels as a function of fold change (FC). For this, the relative expression levels (ratios) between all pairs of genes across all 3 cell lines from the QRT-PCR data were calculated. Values that were filtered out by either Agilent or Amersham were not included in the comparison and thus we did not penalize these 2 platforms for missing values. A total of 3,321 and 3,828 combinations were considered for ratio and single intensity array data, respectively (see Methods). Each gene pair combination was attributed to 1 out of 9 FC bin depending on its QRT-PCR value. We then analyzed the concordance of log_2_-ratio (cell line/REF) array data to -ΔΔCt QRT-PCR data by -ΔΔCt FC bins. To optimize comparisons with single intensity platforms we also compared -ΔCt QRT-PCR values to log_2_-intensity values as a function of -ΔCt FC bins. Figure 5 shows box plots of the distributions of the ratios obtained from array data of the gene pair combinations per FC bin. Although we observe a general trend of array FC levels concordant with the QRT-PCR data, transforming single intensity data into log2-ratios resulted in a marked reduction in the range of array FCs (compare Fig. 5A with D and B with E for Affymetrix and Amersham, respectively). Based on log_2_-ratio array data and -ΔΔCt data we observed a closer relationship of Agilent FCs to QRT-PCR FCs (Fig 5C). We also observed that for extreme FCs calculated based on QRT-PCR (superior to or equal to log_2 _FC of 5.0) 25% of the FCs obtained from Amersham ratio data were below 0. If, however, we base our assessment of single-intensity platforms on single-intensity data we see a much closer correlation with QRT-PRC data, particularly for Affymetrix which yielded a near linear relationship with QRT-PCR data ranging from the minimal to the maximal FC bin (Fig. 5E).

## Conclusion

Our study compares the latest, state-of-the-art short and long oligonucleotide human-microarray commercial technologies. The results presented here were used for the program CIT, a centralized, multi-tumor program as well as for a similar program launched by the Canceropole Ile-de-France (France's cancer research network) in choosing a human genome-based microarray technological platform that can meet a high-throughput demand. Therefore, the design of the study was adapted to judge each company in the context of these types of programs. In this context, our results show that all platforms yielded reproducible and comparable data. Nevertheless, between the 3 technologies we found that one-color platforms were more precise while Affymetrix and Agilent were more concordant based on the agreement of expression measurements and identification of differentially expressed genes. In addition, Affymetrix was found to have a slightly higher sensitivity at detecting relative differences in gene expression levels (irrespective of the magnitude of change) and an overall higher level of concordance with the QRT-PCR data.

## Methods

### RNA samples and number of slides analyzed per platform

Human cell lines Jurkat (T cell acute lymphoblastic leukemia), MCF7 (breast cancer) were provided by INSERM U462, Institute Universitaire d'Hematologie, Hospital Saint Louis, Paris, France) and BattP12 (mesothelioma) was established and kindly provided by Dr. Marie-ClaudeJaurand (Université Paris XII, Paris, France). All cell lines were cultured in RPMI 1640 supplemented with 10% fetal calf serum, 10 U/ml penicillin, 100 μg/ml streptomycin and 2 mM L-glutamine. Cells were harvested during exponential phase growth and total RNAs were extracted as previously described [34]. Universal human reference RNA (REF, Stratagene) was recovered following manufacturer instructions. One hundred micrograms of each RNA were then purified using QIAGEN (QIAGEN S.A., Courtaboeuf, France) RNeasy mini kit, as describe in manufacturer's clean-up column protocol. All RNAs were aliquoted and stored at -80°C. Aliquots of the RNAs were analyzed by electrophoresis on a Bioanalyser 2100 (version A.02 S1292, Agilent Technologies, Waldbronn, Germany) and quantified using Nano Drop™ ND-1000 (Nyxor Biotech). Criteria for qualification was a 28 s/18 s ratio above 1.8, and an optical density at 260 nm and 280 nm ratio above 1.80. Between 2 – 25 μg per cell line along with 3 – 25 μg REF RNA were then sent to each platform (Affymetrix UK Ltd., High Wycombe, United Kingdom; Agilent Technologies, Waldbronn, Germany; GE Healthcare (Amersham), Freiberg, Germany).

### Probe labeling, array hybridizations and image analyses

Barring Agilent, all platforms use a one-color labeling approach. Agilent arrays were co-hybridized with cell line samples and the reference sample. Each platform followed protocols that are publicly available at their respective web sites [35-37]. Briefly, each platform hybridized the following material: 10 μg fragmented cRNA, Affymetrix; 10 μg fragmented cRNA, Amersham; 0.75 μg cRNA labeled with Cy3 (cell line sample) and Cy5 (REF) from 3 independent labeling reaction per cell line, Agilent; 10 μg fragmented cRNA, PX. Raw data from the following were obtained and processed: 12 Affymetrix HG-U133 plus 2.0 GeneChip™ arrays (3 arrays per sample type independently labeled, March, 2004); 12 Agilent's "44 K whole genome" arrays (4 arrays per cell line (3 arrays for 3 independent labeling reactions plus one technical replicate, June 2004); 12 Amersham CodeLink™ UniSet Human 20 K Human arrays (3 slides per sample type labeled in batch, March, 2004); 8 PX arrays (2 slides per sample type labeled in batch, February, 2004). A summary of the relative representation of available public data bank identifiers for each array is given [see Additional file 1]. The annotation information was supplied by each platform at the time the experiment was conducted. Since the time of this analysis GE Healthcare has launched a new CodeLink Human Whole Genome Bioarray which is claimed to target roughly 45,000 well characterized human gene and transcript targets. All raw data and associated annotations are publicly available (acc.# E-MEXP-467) at ArrayExpress [38].

### Data filtering and normalization

Filtering information with the aim of removing data obtained from non-viable targets (spotted or synthesized oligonucleotide) was provided by Amersham, Agilent and PX. Briefly, for Agilent, 20 different criteria were used to flag a specific target based on signal/noise ratio, spot morphology or homogeneity. For Amersham only spots with intensities above the negative control threshold (20% trim-mean of negative controls + 3 standard deviations using the trim-mean set) were included in the analysis. PX filtered targets based on a signal/noise threshold ratio of 3 and those called "not-detected" by their spot-detection software. The criteria used to flag targets were not imposed or suggested but rather derived from the respective platforms. We did not receive pre-processed or filtered data from Affymetrix. The following methods were used to normalize each data sets: robust multi-average (RMA) normalization and expression summary [39] for Affymetrix HG-U133 plus 2.0 raw data; median normalization (BRB-Array Tools v3.2 beta5.0) for Amersham CodeLink and PX raw, filtered data; lowess non-linear normalization [40] for Agilent raw, filtered data. Only available data from each platform were taken into consideration in our evaluation of platform discordance of expression measurements. When necessary, all single-intensity platform data (Affymetrix, Amersham and PX) were transformed into log_2_-ratios using the median value of the universal reference samples as the denominator.

### Intensity quantile bin attribution

For each platform we attributed 1 out of 4 intra-platform intensity quantiles (Q1, ..., Q4) to each available targets out of all available targets per platform. The rank of the median intensities across all 3 cell lines for all targets were used to attribute an intensity quantile bin to a given target. Therefore targets in first quantile (Q1) have the 25% weakest expressed median intensities and Q4 corresponds to the targets having 25% strongest median intensities. We then determined the number of targets having similar inter-platform (consensus) quantile attributions (cQ1, ..., cQ4) between all 4 platforms. Here "similar" refers to a situation where the minimal and maximal quantile attributions to a given target doesn't differ more than 1 (e.g. min = Q1 and max = Q2; min = Q2 and max = Q3). Using the quantile information for each platform 1 of 7 consensus quantiles (cQ, mean of the original quantile attributions) was attributed to each of these targets using the following partitioning: cQ1 for targets with mean quantile attributions (Q_mean_) between 1–1.25; cQ1.5: Q_mean _= 1.5; cQ2: Q_mean _= 1.75 – 2.25; etc.). We also determined the number of targets having "discordant" quantile attributions unique to a given platform (min Q – max Q = 2 while "similar" for 3 out of the 4 platforms) as well as discordant between all platforms (min Q – max Q = 2 and not similar for 3 out of the 4 platforms (see [Additional file 2] for a list of the 3.4 K common RefSeq accession numbers as well as the original quantile and cQ attributions).

### 3.4 K common data set

In order to eliminate the problem of multiplicity (1: N and N: 1 relationships between targets and transcripts), only non-redundant targets (one feature per target ID) in which there was a 1 target: 1 transcript (RefSeq identifier) correspondence represented on the slide were considered (PX: 11,271 targets; Agilent: 10,363 targets; Amersham: 9,964; Affymetrix: 8,705 targets). Sequence-based mapping between the different platforms was not feasible as oligonucleotide sequence information was not available for each of the 4 platforms (only for Affymetrix and Agilent). We used the provided representative sequence identifier (Genbank and RefSeq accession number) to map each oligonucleotide to an Entrez Gene identifier that was available at the time (April 2004). We found that out of the 6,664 unique sequence identifiers that were found in common between the different platforms, 6,404 mapped to Entrez Gene identifiers. Only single transcripts associated with a Entrez Gene identifiers represented only once on the arrays (3,471 targets ("3.4 K set")) were used for direct comparisons of relative expression measurements. This set of 3.4 K targets were representative, at the feature-intensity level, for each array type (25% representation in each intensity-quantile bin (quantiles defined using all targets for each array type).

### Statistical tests

To determine the genes that discriminate the different cell lines we performed independent ANOVA analyses (F tests) and t tests (MCF7 versus Batt P12) for the 3 platforms. All univariate t and F tests using BRB ArrayTools (v3.2 b5) using the log_2 _single-intensity data for Affymetrix and Amersham and log_2_-ratio data obtained for Agilent. "Discriminating targets" were determined using a nominal significance level of each univariate test of p < 0.001. The F test was based on comparing the differences in mean log_2_-intensities (or log_2_-ratios) between the 3 cell lines relative to the variation expected in the mean differences. Due to small sample population for each platform we used a random variance model for the univariate tests when distribution assumptions underlying this model were met [41]. These assumptions were satisfied for all platforms except for F tests conducted for the Affymetrix data (here, we used the within class variance calculated for each target).

### Cluster analysis

For the clustering analysis shown in Figure 2, samples were clustered using R software (v2.0.1; package class v7.2.10) and the following parameters: complete linkage; distant metric = 1 – Pearson correlation (the distance measured between any two samples was calculated using the maximal number of common targets between the two samples). Though other inter-platform normalization methods and clustering methods were tested (z-score, quantile normalization), we found that only by using a common reference sample and transforming single intensity data into a log ratio enabled us to overcome the platform-dependent bias and observe cross-platform cell line-specific clusters.

### Quantitative RT-PCR

Quantitative RT-PCR (QRT-PCR) reactions were performed as previously described [42] for each of 15 genes (see [Additional file 3] for gene list, sequences and position for each primer/probe set) using the same aliquots originally sent to each platform. Briefly, the reverse transcription was performed using 4 μg RNA in a final volume of 80 μl. For each set of primers and probe 2 μL of cDNA was diluted to total volume of 25 μL containing 12.5 μL Universal Master Mix (Applied Biosystems, Foster City, CA, USA), 0.3 μM of each primers and 0.2 μM Taqman^® ^probe. After initial steps for 2 min at 50°C and 10 min at 95°C, 50 cycles at 95°C for15 sec and 60°C for 1 min were performed on an ABI Prism 7700 (Applied Biosystems, Foster City, CA, USA). Primers and Taqman^® ^probes for the selected set of genes were chosen using Primer Express software (Applied Biosystems, Foster City, CA, USA) based on the primer-probe combinations prioritizing the 5' end of the transcript and in which the sense and anti-sense primers corresponded to 2 different exons separated by an intron of at least 500 bases. This yielded several potential combinations. The final combination for each gene was chosen based on thermodynamic criteria. All QRT-PCR reactions included in the analysis yielded amplification curves with ΔRN above 1.0, slopes between -3.1 and -3.8 and correlation coefficients above 0.98. Optimisation of each reaction was based on the performance of a given primer-pair/probe combination and the resulting standard curves from dilution series experiments (1:10 to 1:100,000 of cDNA from samples known to express each gene). Absence of a specific transcript in a given cell line (eg. RAG2 transcript expression in Batt P12 and MCF7 cells and CASP5 transcript expression in Jurkat cells) was assessed based on an absence of amplification in the corresponding QRT-PCR assay. For RAG2 and CASP5 transcripts, sensitivity was experimentally determined to be at 1:10,000 dilution of Jurkat cDNA, and 1:1,000 dilution of peripheral blood mononuclear cell (isolated from blood samples after ficoll purification) cDNA in water, respectively. Based on this dilution series we obtained an optimal slope (-3.68 for Rag2 and -3.43 for CASP5). Normalization of the Ct values was carried out by subtracting the Ct values of the control gene ABL1 yielding ΔCt values for each gene [43]. ΔΔCt values were then derived by subtracting each ΔCt by the ΔCt value obtained from the same gene in the REF sample.

For each cell line, the correlation coefficient (Pearson and Spearman) between each array data sample replicate and the corresponding QRT-PCR data were calculated (see Table 3), using 12 genes out of the 14 (ERBB2 and RPS6KB1 were excluded as there was several corresponding targets in the array data). The R function cor.test was used to calculate the coefficients and associated p values. To measure the concordance of relative expression of the different genes and cell lines two approaches were used. We compared the relative expression of all 14 genes in a given cell relative the reference samples. For this, we compared -ΔΔCt values for each gene in each cell line (42 data points) to the corresponding array ratios obtained as described above. For the all pair-wise gene-gene combination analysis (Fig. 5) we first attributed all the gene-gene ratios calculated across all cell lines based on the -ΔΔCt values calculated from the QRT-PCR data to 9 fold-change bins. From a total number of 4,851 possible gene pair combinations based on the array measurements of 12 out of the 14 genes (excluding ERBB2 and RPS6KB1 and data points (RAG2 and CASP5) not available from QRT-PCR) in the 3 cell lines (3 replicate arrays each) a total number of 3,321 and 3,828 combinations were considered for ratio and single intensity array data, respectively. The distribution of the array data corresponding to each set of combinations were compared by fold change bin. [Additional file 4] gives the sample-matched Ct values as well as for the log_2_-intensities for all genes from each platform.

## Authors' contributions

**AdR**: participated in the conception of analytical design, the analysis of the data and written text; **DG**: QRT-PCR experiments of the 15 genes, primer/probe design; **JMC**: oversaw QRT-PCR experiments of the 15 genes, primer/probe design; **FP**: data re-formatting and submission to EBI; **PD**: mapping of the platform target identifiers to public accession numbers and definition of the number of transcripts corresponding to each target for each platform, participated in the evaluation and interpretation of the results; **FS**: Director of the CIT program; initiated the comparison and participated in the evaluation and interpretation of the results; **DSR**: guided and participated in the analysis and conception of the analytical design, responsible for the majority of the written text; liaison between each platform and all authors.

## Supplementary Material

Additional File 2 **Table of the of 3,471 common target set**. Shown in the table for each of the 3,471 common targets RefSeq identifiers (CommonREFSEQ_4platform) are the HUGO gene symbol (Gene Symbol) and Entrez Gene public data bank identifiers. Also given are the intra-platform intensity quantile attribution calculated for each platform respectively (PX Q, AFFYMETRIX.Q, AGILENT.Q, AMERSHAM.Q) as well as the consensus quantile (cQ) calculated based on the mean quantile attribution obtained for each platform. ANOVA targets (p < 0.001) are indicated for each platform ("1") where "0" indicates a target not filtered and not an ANOVA target. AMER_MCF_BATT, AFFY_MCF_BATT, AGILENT_MCF_BATT indicates the targets that passed the p < 0.001 filter for the respective t tests comparing the MCF7 and Batt P12 samples (see Figure 4 and Tables 1 and 2). Filtered log_2_-intensities are given for each slide hybridized by each platform (PX, Affymetrix (AFFY), Amersham (Amer) or the filtered log_2_-ratios obtained for Agilent (Agilent44K). "NA" is shown for all empty fields corresponding to filtered data points. Click here for file

Additional File 5 **Cluster analysis using the common ANOVA targets**. Sample cluster dendrograms of all 36 platforms samples alone (**A**) or with other data including 66 tumor samples (**B**) and gene cluster dendrogram with associated heat map based on the expression profile of 1,096 (A) common ANOVA targets (or 925 U133A targets mapped to the 1,096 targets for B) found between Affymetrix, Agilent and Amersham. Mapping to the Affymetrix U133A array was based on RefSeq identifiers. Clustered sample groups corresponding to the cell line and similar tumor type are indicated by colored bars. Log_2_-ratios were centered and color coded (below heat map B). MCF-7 samples are represented with blue bars, BattP12 and mesothelioma samples are represented with red bars while Jurkat and TALL samples are represented by green bars. BattP12 is a mesothelioma cell line and clusters with the MESO samples (red bar) which as a group clusters with the MCF7 samples (blue bar). Jurkat is a T-cell acute lymphoblastic leukemia and clusters with T-ALL samples (green bar). As a part of the CIT program we obtained raw Affymetrix HG-U133A data for 66 samples corresponding to either pooled tumors of a particular cancer type and/or derived cell lines (unpublished data). These samples include the universal reference RNA from Stratagene (see above). Tissues represented are as follows: B-cell acute lymphoblastic leukemia (BALL, n = 4); colon cell line (COLO, n = 8); follicular lymphoma (FOLL, n = 4); hepatocarcinoma (FOIE, n = 3); myeloblastic acute leukemia (LAM, n = 3); epidermotrophal lymphoma (LEPI, n = 2); chronic lymphoblastic leukemia (LLC, n = 1); myeloblastic chronic leukemia (LMC, n = 2); mesothelioma cell line (MESO, n = 8); epithelial cells (PEAU, n = 4); lung (POUM n = 3); T-cell acute lymphoblastic leukemia T (TALL, n = 20); thyroid (THYR n = 4). Labeled cRNA reactions, hybridizations and image analyses for all 66 samples were carried out at the IGBMC, Strasbourg, France (March, 2002). Raw data was normalized and summarized expression values were generated using RMA (described above). For all cluster analyses, normalized intensity values were transformed into log_2_-ratios using the reference sample as the denominator. Cluster analysis was performed using log_2 _ratios (ratios calculated as described above) and DNA-Chip Analyzer (dChip) Version 1.3, with the following parameters: distant metric = 1-pearson correlation; linkage = centroid; row standardization. Click here for file

Additional File 4 **Array and QRT-PCR data for the 15 genes analyzed by QRT-PCR**. This table is divided into three sections. Section I shows the filtered log_2 _intensities (Affymetrix and Amersham) or log_2_-ratios (Agilent) for the 15 genes analyzed by QRT-PCR. RNA batches are indicated above each table. Multiple targets were available for ERBB2 and RPS6KB1. Section II shows Ct values for each RNA batch obtained for each of the 2 batches (RNA batches are indicated above each table where Batch 1: green; Batch 2: pink). For sections I and II A red X is given for data points that were either filtered out by a given platform (microarray data) or for genes that were below the level of detection in a given cell line determined by QRT-PCR. Section III shows the number of possible versus available gene pair combinations QRT-PCR fold change bins used in Figure 5. The number of possible gene pair combinations per fold change bin were based on the distribution of relative expression levels calculated using the -ΔΔCt values to compare with log_2_-ratio array data (cell line/reference for all 3 platforms, shown in yellow) or using -ΔCt values to compare with log_2 _intensities (from Amersham and Affymetrix, shown in blue). Nine different bins were created with the following code (in bold) corresponding the following log_2_-ratios (x) intervals: <**-5**: x < -5; **-3**: -5 = x < -1.25; **-1**: -1.25 = x < -0.75; **-0.5**: -0.75 = x < -0.25; **0**: -0.25 = x < 0.25; **0.5**: 0.25 = x > 1.25; **1**: 0.25 = x > 1.25; **3**: 1.25 = x > 5.0; > = **5**: x = 5.0. Click here for file

Additional File 1 **Description of the 3 array formats used in the comparisons**. The numbers represent the counts made of total number of targets represented on the respective microarrays. ^1 ^The number of unique public sequence identifiers (ID) represented on the respective microarrays. These counts are based on the annotation table provided at the time the experiments were carried out (March – June, 2004). Control targets were excluded from the counts. Click here for file

Additional File 3 **Primer/Probe information for the 15 genes analyzed by QRT-PCR**. Shown in the table are the 15 genes (Genes Names), accession number, forward and reverse primers and probes with associated 5'-3' sequence, 5' position and size analyzed by QRT-PCR. Click here for file

## Figures and Tables

**Table 1:** Summary of discriminating targets

	**Platform**	**Total**	**Q1**	**Q2**	**cQ**	**< cQ2**
	
**ANOVA**	Affymetrix	1,696	68	140	972	36
	Agilent	1,781	69	325	998	34
	Amersham	1,727	130	414	998	33
**t test (MCF7 vs Batt P12)**	Affymetrix	1,340	54	128	740	36
	Agilent	1,398	48	235	744	23
	Amersham	1,290	79	287	755	24

"Total" refers to the number of discriminating targets out of the 3.4 K data set that were found for each platform based on an ANOVA test of the 3 cell lines or a t test comparing MCF7 and Batt P12 samples. "Q1" and "Q2" refer to lowest 2 intra-platform intensity quantiles. "cQ" (consensus quantile) refers to the number of discriminating targets attributed with similar expression levels across all 4 platforms. The last column refers to the number of discriminating targets with a cQ less than 2.

**Table 2:** Summary of discordance of discriminating targets

		**Uniquely found by a platform**^1^		**Uniquely not found by a platform**^2^	
	**Platform**	**total**	**p > 0.05**	**total**	**p > 0.05**
	
**ANOVA**	Affymetrix	80 (4.7%)	33 (1.9%)	145	44
	Agilent	112 (6.3%)	24 (1.3%)	158	56
	Amersham	171 (9.9%)	47 (2.7%)	228	157
**t test (MCF7 vs Batt P12)**	Affymetrix	72 (5.4%)	13 (1.0%)	178	83
	Agilent	222 (16%)	76 (5.4%)	266	114
	Amersham	190 (15%)	66 (5.1%)	249	56

^1 ^Number of discriminating targets out of the 3.4 K set unique to platform which were available (not filtered) for all platforms (percentage relative to the total number of discriminating targets); of those the number (and percentage) that yielded a p value superior to 0.05 for each of the 2 other platforms. ^2 ^The number of targets uniquely not found to be discriminating and which were available (not filtered) for that platform (including the number of targets with a p value superior to 0.05 for that platform (percentage relative to the total of the uniquely not discriminating targets).

**Table 3:** Correlation of platform data and sample-matched QRT-PCR data

		**Log_2_-ratio vs. -ΔΔCT**				**log_2_-int vs. -ΔCT**			
		**Pearson**	**p**	**Spearman**	**p**	**Pearson**	**p**	**Spearman**	**p**
		
**AFFYMETRIX**	med	0.91	0.001	0.87	0.017	0.79	0.031	0.84	0.016
	max	1		0.97		0.93		0.97	
	
**AMERSHAM**	med	0.72	0.105	0.54	0.297	0.77	0.029	0.9	0.027
	max	0.92		0.88		1		1	
	
**AGILENT**	med	0.92	0.039	0.76	0.208				
	max	1		1					

Shown are median (med) and maximum (max) Pearson and Spearman rank correlation coefficients calculated from all pair-wise combinations between all replicate arrays and for all cell lines. The median p values (p) from all of the tests are also shown. We are presenting 2 types of comparisons depending on the type transformation used: log_2_-ratios platform data versus (vs.) -ΔΔCt QRT-PCR data; and log_2_-intensities (for the one-color platforms) versus -ΔCt QRT-PCR data. Only array data points for 12 out of the 14 genes that were available for all platforms were included in the calculations.

**Table 4:** Gene by gene concordance of relative expression levels between platform data and sample-matched QRT-PCR data

	**QRT-PCR (avg. Ct)**			**Affymetrix**			**Agilent**			**Amersham**		
**GENE**	**B**	**J**	**M**	**B**	**J**	**M**	**B**	**J**	**M**	**B**	**J**	**M**
CASP5	34.03	X	37.85	0	0	0.3	0	0	1	**0.3**	**1**	**0**
RAG2	X	26.36	X	0	1	0	1	1	1	**1**	**1**	**1**
TMPRSS5	30.82	31.70	36.19	1	0.7	0	0	1	1	**1**	**1**	**0**
WNT10B	28.33	28.20	27.32	**1**	**1**	**1**	0.8	0.8	1	0.7	0	1
MAPK4	25.07	29.70	26.83	1	0	0	**0.3**	**1**	**1**	0	1	1
CXCL10	24.58	35.37	32.26	**1**	**0.3**	**0**	1	_	_	1	_	_
RPS6KB1	24.36	23.68	20.72	**1**	**1**	**1**	**1**	**1**	**1**	**1**	**1**	**1**
RME8	22.84	22.17	23.29	1	1	0	**1**	**1**	**0.5**	0	0.3	0
ALG8	22.64	21.75	22.40	**0**	**1**	**0.7**	0	0	0	0	0	0
THEM2	22.62	22.21	22.50	**1**	**1**	**1**	0	0	0	1	1	0.7
ERBB2	22.61	27.45	21.51	1	0.5	1	0.8	0.6	0.9	**1**	**1**	**1**
CCNB2	22.29	20.77	21.93	**0**	**1**	**1**	0	1	0.8	**0**	**1**	**1**
RAB17	22.27	34.69	23.78	**1**	**1**	**1**	1	1	1	1	_	1
BCAS	22.27	21.27	20.60	**0**	**1**	**1**	0	0	1	**0**	**1**	**1**
**Score**				**27.5**			**25.5**			**26**		

The average Ct values (avg. Ct) for each of the 14 genes (the control gene ABL1 is not shown) were calculated from the Ct values obtained from all sample-matched QRT-PCR reactions per cell line (n = 3, BattP12 (B); Jurkat (J); MCF7 (M)). Lower Ct values indicate relatively higher expression levels (see supplemental Table 3 for all Ct values obtained for all 15 genes and for each RNA aliquot). We are reporting the fraction of replicate expression values, by gene and by cell line, from the array data that are over- (or under) expressed in agreement with -ΔΔCt QRT-PCR data (where a score of 1 indicates a perfect concordance). More precisely, the score corresponds to the number of "same direction" (over or under-expressed) expression ratios from array data and QRT-PCR data for a given gene obtained from a given cell line relative to the reference sample divided by the total number of ratios. Data points not detected by QRT-PCR were taken into consideration into the calculation (i.e. if the gene was not detected by QRT-PCR for a cell line the score corresponded to the number of times this data point was filtered by the platform). Dashes indicate filtered data points for all replicate arrays for a given cell line. Fractions in bold indicate the platform yielding the highest sum of the individual fractions for a given gene and a given cell line. Multiple platforms can yield the highest sum for a given gene. The sums of all values for a each platform are given in the last line of the table.
