# Supplementary figures and images for: Comparison of the latest commercial short and long oligonucleotide microarray technologies
Source: BMC Genomics. 2006 Mar 15;7:51. doi: 10.1186/1471-2164-7-51 (PMC1473202; doi:10.1186/1471-2164-7-51)

A.

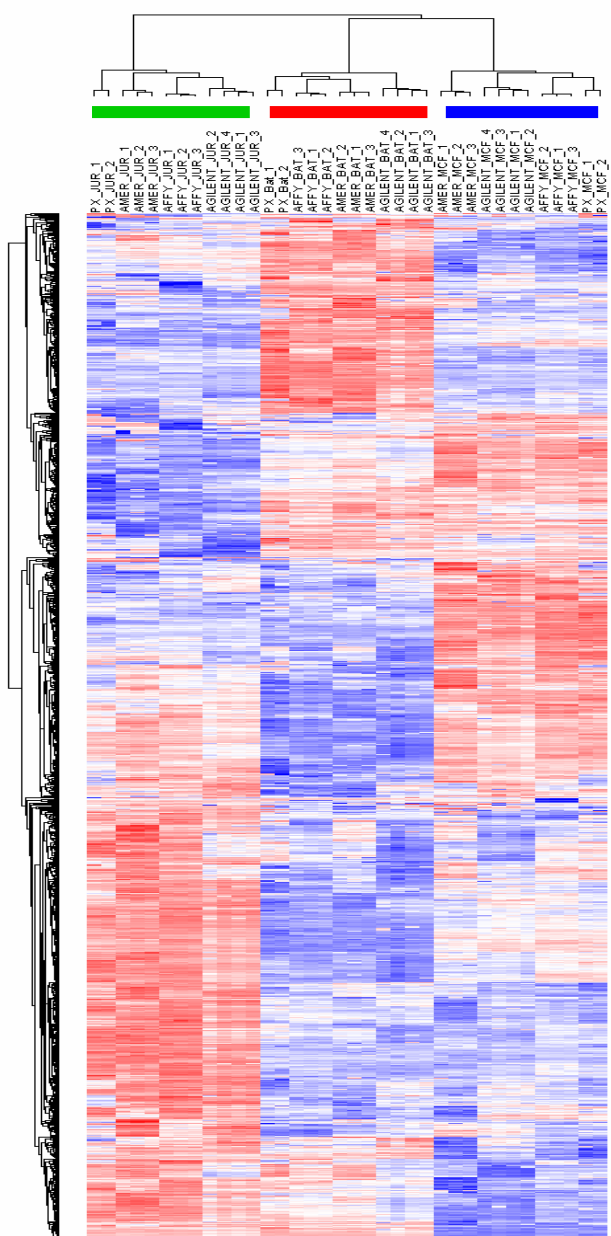

■ Jurkat/TALL  
■ BattP12/MESO  
■ MCF-7

B.

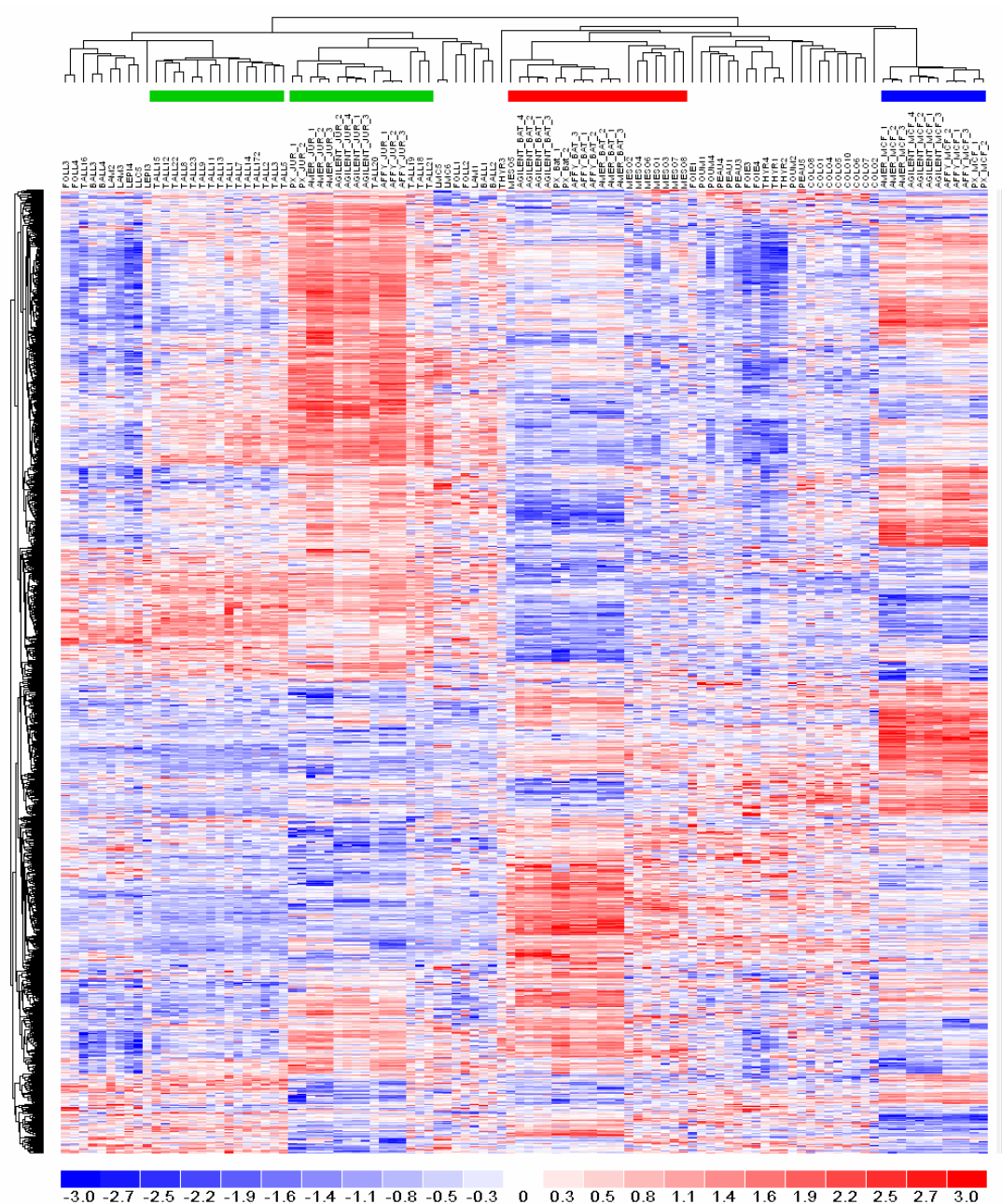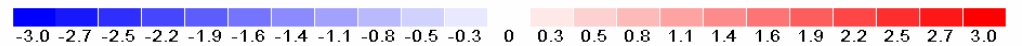

Supplement: Additional File 5 — Cluster analysis using the common ANOVA targets. Sample cluster dendrograms of all 36 platforms samples alone (A) or with other data including 66 tumor samples (B) and gene cluster dendrogram with associated heat map based on the expression profile of 1,096 (A) common ANOVA targets (or 925 U133A targets mapped to the 1,096 targets for B) found between Affymetrix, Agilent and Amersham. Mapping to the Affymetrix U133A array was based on RefSeq identifiers. Clustered sample groups corresponding to the cell line and similar tumor type are indicated by colored bars. Log2-ratios were centered and color coded (below heat map B). MCF-7 samples are represented with blue bars, BattP12 and mesothelioma samples are represented with red bars while Jurkat and TALL samples are represented by green bars. BattP12 is a mesothelioma cell line and clusters with the MESO samples (red bar) which as a group clusters with the MCF7 samples (blue bar). Jurkat is a T-cell acute lymphoblastic leukemia and clusters with T-ALL samples (green bar). As a part of the CIT program we obtained raw Affymetrix HG-U133A data for 66 samples corresponding to either pooled tumors of a particular cancer type and/or derived cell lines (unpublished data). These samples include the universal reference RNA from Stratagene (see above). Tissues represented are as follows: B-cell acute lymphoblastic leukemia (BALL, n = 4); colon cell line (COLO, n = 8); follicular lymphoma (FOLL, n = 4); hepatocarcinoma (FOIE, n = 3); myeloblastic acute leukemia (LAM, n = 3); epidermotrophal lymphoma (LEPI, n = 2); chronic lymphoblastic leukemia (LLC, n = 1); myeloblastic chronic leukemia (LMC, n = 2); mesothelioma cell line (MESO, n = 8); epithelial cells (PEAU, n = 4); lung (POUM n = 3); T-cell acute lymphoblastic leukemia T (TALL, n = 20); thyroid (THYR n = 4). Labeled cRNA reactions, hybridizations and image analyses for all 66 samples were carried out at the IGBMC, Strasbourg, France (March, 2002). Raw data was normalized and [file 1471-2164-7-51-S5.pdf]
